# A novel hybrid method based on task-related component and canonical correlation analyses (H-TRCCA) for enhancing SSVEP recognition

**DOI:** 10.3389/fnins.2025.1544452

**Published:** 2025-04-25

**Authors:** Amin Besharat, Nasser Samadzadehaghdam, Tahereh Ghadiri

**Affiliations:** ^1^Department of Biomedical Engineering, Faculty of Advanced Medical Sciences, Tabriz University of Medical Sciences, Tabriz, Iran; ^2^Department of Neuroscience and Cognition, Faculty of Advanced Medical Sciences, Tabriz University of Medical Sciences, Tabriz, Iran

**Keywords:** brain-computer interface (BCI), electroencephalogram (EEG), steady-state visual evoked potentials (SSVEP), canonical correlation analysis (CCA), task-related component analysis (TRCA), spatial filtering

## Abstract

**Introduction:**

Brain-computer interfaces (BCIs) based on steady-state visual evoked potentials (SSVEP) rely on the brain’s response to visual stimuli. However, accurately recognizing target frequencies using training-based methods remains challenging due to the time-consuming calibration sessions required by subject-specific training methods.

**Method:**

To address this limitation, this study proposes a novel hybrid method called Hybrid task-related component and canonical correlation analysis (H-TRCCA). In the training phase, four spatial filters are derived using canonical correlation analysis (CCA) to maximize the correlation between the training data and reference signals. Additionally, a spatial filter is also computed using task-related component analysis (TRCA). In the test phase, correlation coefficients obtained from the CCA method are clustered using the k-means++ clustering algorithm. The cluster with the highest average correlation identifies the candidate stimuli. Finally, for each candidate, the correlation values are summed and combined with the TRCA-based correlation coefficients.

**Results:**

The H-TRCCA algorithm was validated using two publicly available benchmark datasets. Experimental results using only two training trials per frequency with 1s data length showed that H-TRCCA achieved average accuracies of 91.44% for Dataset I and 80.46% for Dataset II. Additionally, it achieved maximum average information transfer rates of 188.36 bits/min and 139.96 bits/min for Dataset I and II, respectively.

**Discussion:**

Remarkably H-TRCCA achieves comparable performance to other methods that require five trials, utilizing only two or three training trials. The proposed H-TRCCA method outperforms state-of-the-art techniques, showing superior performance and robustness with limited calibration data.

## 1 Introduction

In recent years, brain-computer interfaces (BCIs) have become increasingly important in various applications, enabling individuals with disabilities or neurological disorders to communicate directly with their external environment, without relying on peripheral nerves and muscles (Aghdam et al., 2020; Meng et al., 2024). Among different BCI approaches, SSVEP-based brain communication technology has gained significant attention from researchers due to its advantages, including high accuracy, high information transfer rate (ITR), high signal-to-noise ratio (SNR), and minimal user training requirements (Nguyen and Chung, 2018; Lee and Choi, 2021). The brain produces an oscillatory electrical potential known as SSVEP when an individual directs their attention to a periodic visual stimulus within the frequency range of 4–60 Hz (Alimardani et al., 2018). This electrical activity is detected in the occipital region at frequencies corresponding to the stimulus or its higher harmonics (Zhang et al., 2020).

Recently, various algorithms have been proposed for recognizing SSVEP frequencies. These algorithms can be categorized as calibration-free and calibration-based approaches, depending on the availability of calibration data (Zerafa et al., 2018; Besharat et al., 2024). Calibration-free methods do not rely on training data to extract SSVEP features. Instead, they use a classifier, usually based on the correlation coefficient value, to select the stimulus with the highest feature value, which is expected to elicit the strongest SSVEP response (Zerafa et al., 2018). One commonly used calibration-free approach is canonical correlation analysis (CCA), which aims to find weights that maximize the correlation between SSVEP signals and reference signals (sine-cosine) (Lin et al., 2006). Nevertheless, CCA has several limitations, including its sensitivity to noise, phase shifts, constraints on the time window length, and underutilization of harmonic components, all of which can negatively affect the accuracy of frequency recognition (Besharat et al., 2024). Several studies have been proposed to overcome these limitations and improve its performance. These studies have introduced improved methods, such as filter bank CCA (FBCCA) (Chen et al., 2015), filter bank temporally local CCA(FBTCCA) (Shao and Lin, 2020), spatio-spectral CCA (SSCCA) (Cherloo et al., 2022), and CCA based on signal extension (SE-CCA) (Li et al., 2023).

Several extended variants of conventional CCA have been proposed to optimize predefined reference signals to enhance performance (Zhang et al., 2011; Zhang et al., 2013; Ziafati and Maleki, 2022). Zhang et al. (2014) introduced the Multiset CCA (MsetCCA) technique, which enhances SSVEP frequency recognition by optimizing reference signals through multiple linear transforms. Wong et al., (2020a) introduced MsetCCA-R, an improved version of MsetCCA, aiming to boost SNR by minimizing non-SSVEP-related elements in EEG signals. Leveraging additional insights from sine-cosine reference signals, MsetCCA-R excels in SSVEP recognition tasks, enhancing target detection accuracy in BCIs.

Recent studies highlight significant improvements in classification performance, particularly in detecting stimulus frequencies, in calibration-based methods compared to calibration-free methods (Zhang et al., 2022; Apicella et al., 2023; Hamou et al., 2024). Among these, task-related component analysis (TRCA) and its improved versions have gained considerable attention. The TRCA method, introduced by Nakanishi et al. (2017), stands out for its superior performance over other spatial filtering methods. It achieves task-related component extraction by optimizing the inter-trial covariance among individual training data. Wong et al., (2020b) introduced a novel approach to mitigate the underestimation of the covariance matrix in TRCA when there is a lack of sufficient training data. This technique involves leveraging data from multiple stimuli, including both the target and its neighboring stimuli, to expand the training set. Their research demonstrated that this multi-stimulus TRCA (msTRCA) method significantly outperformed the TRCA. Wong et al. (2020) proposed the TRCA with a sine-cosine reference signal (TRCA-R) algorithm, which enhances performance compared to TRCA, especially with small training datasets. This approach involves projecting the EEG data into a subspace defined by reference signals. The projection is carried out using an orthogonal projector derived from the QR factorization of these reference signals. Another study by Sun et al. (2021) introduced the scTRCA (similarity-constrained TRCA) algorithm to enhance SSVEP detection in BCIs. By using sine-cosine templates, this method filters out task-related noise, ensuring the extracted components are reproducible across trials and highly correlated with SSVEPs. The algorithm constructs an optimal spatial filter through a constrained optimization problem, showing significant improvements, especially with limited training data. Oikonomou (2022) introduced adaptive TRCA (adTRCA), which is a spatial filtering method that utilizes temporal data from EEG trials. This method employs multitask learning within a Bayesian framework to integrate temporal information into the overall procedure. In another study, Huang et al. (2022) introduced latency aligning TRCA (LA-TRCA), a technique aimed at improving SSVEP-based BCIs by aligning visual latencies across channels to accurately extract phase information from task-related signals. Subsequently, TRCA is employed for frequency detection on the aligned data epochs. Recently, Zhou et al. (2024) proposed TRCA Dynamic Window deep Q-network (TRCA-DW-DQN), a method for SSVEP detection that dynamically adjusts the window length based on signal features, unlike the fixed window length in traditional TRCA. This flexibility enhances speed, accuracy, and overall performance across various conditions and subjects. Kumar and Reddy (2019) presented the sum of squared correlation (SSCOR) algorithm, which optimizes the sum of squared correlations among inter-session individual data to create template signals. In another study, Wei et al. (2020) proposed a method called training data-driven CCA (TDCCA) that aims to enhance the robustness of spatial filters. They achieved this by training the filters using a correlation between concatenated training data and individual templates. Recently, Yuan et al. (2022) introduced an innovative framework to enhance frequency recognition. This method uses CCA to train two spatial filters obtained by using concatenated individual training data and reference signals, demonstrating significant advancements in frequency recognition.

Despite the promising results achieved by the algorithms in detecting SSVEP frequencies, there is still potential for further improvement. Most of these algorithms perform better when evaluated with sufficient calibration data, yet their effectiveness diminishes when the training data is limited. Therefore, the ability to achieve high classification performance with a small number of training data sets becomes more crucial in BCI applications. In this study, we propose a novel hybrid approach known as Hybrid task-related component and canonical correlation analysis (H-TRCCA) that aims to improve the performance of SSVEP recognition using a limited number of training trials. H-TRCCA is inspired by the techniques of CCA and TRCA. The H-TRCCA method involves deriving five spatial filters from the training data and an artificial signal through correlation analysis and covariance maximization. During the test phase, the stimuli with the highest correlation coefficients are identified using the k-means++ method. Subsequently, the correlation coefficients obtained from CCA-based spatial filters are combined with the correlation coefficients obtained from TRCA for each candidate stimulus. The H-TRCCA method was evaluated on the benchmark and BETA datasets, and its performance was compared to the MsetCCA-R, SSCOR, TRCA, and msTRCA algorithms.

## 2 Materials and methods

### 2.1 Data description

In this study, we utilized two publicly accessible datasets: the benchmark dataset (Wang et al., 2016) and the BETA dataset (Liu et al., 2020).

The benchmark dataset (Dataset I) contains EEG data from 35 healthy individuals (17 females, aged 17–34 years, mean age: 22 years). Among these participants, 8 (S01-S08) had prior experience, while the remaining 27 (S09-S35) were naive to the experiment. The dataset included 40 target characters; each assigned a distinct stimulus frequency ranging from 8 Hz to 15.8 Hz at intervals of 0.2 Hz. Each subject’s dataset was divided into six blocks, with 40 trials (representing stimuli) within each block, resulting in 240 recorded trials. The duration of each trial spanned 6 s: 0.5 s pre-stimulus, 5 s for visual stimulation, and the last 0.5 s for a blank screen.

The BETA dataset (Dataset II) comprises EEG recordings from 70 healthy users, including 28 females and 42 males, with an average age of 25.14 years. The experiments in this dataset were conducted in a non-laboratory environment, leading to a lower SNR. It includes 40 target characters and consists of 4 blocks per experiment. Each subject’s dataset consists of a total of 160 recorded data trials. For 15 subjects, each trial has a duration of 3 s and follows this timing structure: 0.5 s before stimulation onset, 2 s for visual stimulation, and 0.5 s after stimulation. For the remaining 55 subjects, the visual stimulation duration is consistent at 3 s, while the pre- and post-stimulation periods follow the same structure as the group of 15 subjects.

Both datasets have 64 channels, based on the international 10–20 system, and were recorded at a sampling frequency of 250 Hz. A 50 Hz notch filter was applied to eliminate power-line interference during EEG recording. In this study, we conducted SSVEP signal analysis using nine channels (Pz, PO5, PO3, POz, PO4, PO6, O1, Oz, and O2). The selection of SSVEP signals was based on a time range of [0.14 s, 0.14+ d s], as defined in the benchmark dataset, and [0.13 s, 0.13+ d s], as defined in the BETA dataset, where d represents the time window used in the analysis (Wang et al., 2016; Liu et al., 2020).

The two datasets differed in their experimental conditions: Dataset I was recorded with electromagnetic shielding, while Dataset II was recorded without electromagnetic shielding.

### 2.2 Canonical correlation analysis (CCA)

Standard CCA is a multivariate statistical technique to find correlations between two sets of variables (Lin et al., 2006; Maye et al., 2017). For SSVEP frequency recognition, CCA uses two multivariate variables, namely, multi-channel EEG signals $X\in {\mathbb{R}}^{N_{c}\times N_{s}}$ and reference signals (sine-cosine) $Y_{n}\in {\mathbb{R}}^{2N_{h}\times N_{s}}$ to find the maximum correlation. Where $N_{c}$ , $N_{s}$ , and $N_{h}$ denote the number of channels, the number of data samples (data length), and the number of harmonics. In the standard CCA algorithm, the reference signals are set to be the series of sine-cosine waveforms of the $n^{th}$ stimulation frequency $f_{n}$ and its harmonics, that are constructed according to the following Equation 1 for each visual stimulus.


(1) $$Y_{n}=\left[\begin{array}{c} \sin\left(2\pi f_{n}t\right)\\ \cos\left(2\pi f_{n}t\right)\\ .\\ .\\ .\\ \sin\left(2\pi N_{h}f_{n}t\right)\\ \cos\left(2\pi N_{h}f_{n}t\right) \end{array}\right],t=\left[\frac{1}{f_{s}},\frac{2}{f_{s}},\ldots ,\frac{N_{s}}{f_{s}}\right]$$

where $f_{s}$ refers to the sampling rate. The purpose of this algorithm is to find the weight vectors $w_{x}\in {\mathbb{R}}^{N_{c}\times 1}$ and $v_{y}\in {\mathbb{R}}^{2N_{h}\times 1}$ that maximize the correlation between the canonical variables $w_{x}^{T}X$ and $Y_{n}^{T}v_{y}$ (linear combination of reference signal harmonics), as shown in Equation (2):


(2) $$\rho_{n}\left(x,y\right)=\underset{w_{x},v_{y}}{\arg\;\max}\frac{E\left[w_{x}^{T}XY_{n}^{T}v_{y}\right]}{\;\sqrt{E\left[w_{x}^{T}XX^{T}w_{x}\right]\;E\left[v_{y}^{T}Y_{n}Y_{n}^{T}v_{y}\right]}}$$

The target stimulus $f_{t}$ is determined by the stimulant frequency that exhibits the highest correlation with the EEG signals, as shown in Equation (3):


(3) $$f_{t}=\underset{n}{\mathrm{argmax}}\rho_{n},\;n=1,2,\ldots ,N_{f}$$

where $N_{f}$ indicates the number of stimulation frequencies.

### 2.3 Filter-bank analysis

The filter-bank strategy aids in target stimuli classification by decomposing SSVEPs into sub-band components and extracting independent information from the harmonic components, thereby enhancing the SNR for improved classification (Chen et al., 2015). In this study, Type I Chebyshev IIR digital filters were employed to create five sub-bands (i.e., 8* b -90 Hz, b ∈ [1, 5]) for all the frequency detection techniques as data preprocessing. These filters were utilized with passbands/stopbands set at [6/4, 14/10, 22/16, 30/24, 38/32 Hz], and low-pass bands/stopbands set at [90/100 Hz] (Lan et al., 2023). The overall detection score is determined by taking a weighted sum of the squared feature values across all sub-bands, as shown in Equation (4):


(4) $$\rho_{n}=\overset{N_{b}}{\underset{b=1}{\Sigma }}c\left(b\right).{\left({\hat{r}}_{n}^{b}\right)}^{2}$$

where ${\hat{r}}_{n}^{\left(b\right)}$ denotes the feature extracted from the $b^{th}$ sub-band and $c\left(b\right)=b^{-1.25}+0.25$ is the weight function defined in Kumar and Reddy (2019).

### 2.4 Proposed method

In this study, we propose the H-TRCCA algorithm, which combines CCA and TRCA to generate five different spatial filters from the training data and reference signal. The CCA method produces four types of spatial filters, while a single spatial filter is derived using the TRCA method.

#### 2.4.1 Training step

First and second spatial filters – CCA method.

Using the CCA method, we calculate the first and second spatial filters by assessing the correlation between the concatenated sine-cosine reference signals denoted by $Y_{n,ref}=\left[Y_{n},Y_{n},\ldots Y_{n}\right]\in {\mathbb{R}}^{2\times N_{h}\times \left(N_{s}*N_{t}\right)}$ , and the concatenated individual template $K_{n}=\left[{\bar{X}}_{n},{\bar{X}}_{n},\ldots ,{\bar{X}}_{n}\right]\in {\mathbb{R}}^{N_{c}\times \left(N_{s}*N_{t}\right)}$ for the $n^{th}$ stimulus frequency. The template is derived by averaging multiple training trials $X_{n}^{t}\in {\mathbb{R}}^{N_{c}\times N_{s}}$ , represented as ${\bar{X}}_{n}=\left(\frac{1}{N_{t}}\right)\overset{N_{t}}{\underset{t=1}{\Sigma }}X_{n}^{t}$ , where $N_{t}$ represents the number of training trials (Wang et al., 2014). The optimization problem is formulated as shown in Equation (5):


(5) $$\rho_{1}=\underset{w_{x},v_{y}}{\arg\;\max}\frac{E\left[w_{x}^{T}K_{n}Y_{n,ref}^{T}v_{y}\right]}{\sqrt{E[w_{x}^{T}K_{n}K_{n}^{T}w_{x}}]\;\sqrt{E\left[v_{y}^{T}Y_{n,ref}Y_{n,ref}^{T}v_{y}\right]}}$$

The weight vectors, denoted as $w_{x}$ and $v_{y}$ , are adopted as spatial filters for feature extraction and subsequently renamed as $w_{a}$ and $w_{b}$ , respectively.

Third and fourth spatial filters – CCA method.

To obtain the third and fourth spatial filters, the correlation between the training data $X_{n}=\left[X_{n}^{1},X_{n}^{2},\ldots ,X_{n}^{N_{t}}\right]\in {\mathbb{R}}^{N_{c}\times \left(N_{s}*N_{t}\right)}$ (formed by concatenating $N_{t}$ training trials) and the $Y_{n,ref}$ (Yuan et al., 2022) is calculated using the provided Equation 6:


(6) $$\rho_{2}=\underset{w_{x},v_{y}}{\arg\;\max}\frac{E\left[w_{x}^{T}X_{n}Y_{n,ref}^{T}v_{y}\right]}{\sqrt{E[w_{x}^{T}X_{n}X_{n}^{T}w_{x}}]\;\sqrt{E\left[v_{y}^{T}\;Y_{n,ref}Y_{n,ref}^{T}v_{y}\right]}}$$

The weight vectors, denoted as $w_{x}$ and $v_{y}$ , are adopted as spatial filters for feature extraction and subsequently renamed as $w_{c}$ and $w_{d}$ , respectively.

Fifth spatial filter – TRCA method.

The fifth spatial filter is obtained using the TRCA method, which extracts task-related components in SSVEP-based BCIs by maximizing inter-trial covariance (Nakanishi et al., 2017). The corresponding optimization process involves simplifying the maximization of the sum of covariances. This simplification is done by expressing the problem as a Rayleigh-Ritz eigenvalue problem, as shown in Equation (7):


(7) $$\hat{w}=\underset{w}{\mathrm{argmax}}\frac{w^{T}Sw}{w^{T}Qw}$$

where S and Q are the sum of cross-covariance and the sum of auto-covariance matrices, respectively, as defined in Equation (8):


(8) $$Q=\overset{N_{t}}{\underset{\begin{array}{c} i,j=1\\ i\neq j \end{array}}{\Sigma }}cov\left(X_{i},X_{j}\right)\text{ and }S=\overset{N_{t}}{\underset{\begin{array}{c} i,j=1\\ i=j \end{array}}{\Sigma }}cov\left(X_{i},X_{i}\right)$$

where i and j denote the indices of trials, $X_{i}\in {\mathbb{R}}^{N_{c}\times N_{s}}$ is the single trial data. The weight vector, denoted as $\hat{w}$ , can be obtained as the eigenvector of matrix $Q^{-1}S$ corresponding to its largest eigenvalue, which is subsequently referred to as $w_{t}$ .

#### 2.4.2 Test step

The testing phase consists of two stages to determine the target stimulus. In the first stage, the stimuli with the highest probability of being the target stimuli are identified and selected as candidates. Subsequently, one stimulus is chosen from the candidate stimuli as the final target stimulus.

In the first stage, given a single-trial test signal $X\in {\mathbb{R}}^{N_{c}*N_{s}}$ , the canonical correlation between X and $Y_{n}$ and four Pearson’s correlation coefficients between the projected signals of $X^{T}w_{a}$ and ${\bar{X}}_{n}w_{a}$ , $X^{T}w_{a}$ and $Y_{n}w_{b}$ , $X^{T}w_{c}$ and $Y_{n}w_{d}$ , $X^{T}w_{c}$ and ${\bar{X}}_{n}w_{c}$ are calculated, as formulated in Equation (9):


(9) $${\hat{r}}_{n}=\left[\begin{array}{c} \begin{array}{c} \begin{array}{c} {\hat{r}}_{n}\left(1\right)\\ {\hat{r}}_{n}\left(2\right)\\ {\hat{r}}_{n}\left(3\right) \end{array}\\ {\hat{r}}_{n}\left(4\right) \end{array}\\ {\hat{r}}_{n}\left(5\right) \end{array}\right]=\left[\begin{array}{c} \begin{array}{c} \rho (X^{T}w_{x}\left(X,Y_{n}\right),Y_{n}w_{Y}\left(X,Y_{n}\right)\\ \rho (X^{T}w_{a}\left(K_{n},Y_{n,ref}\right),{\bar{X}}_{n}w_{a}\left(K_{n},Y_{n,ref}\right)\\ \rho (X^{T}w_{a}\left(K_{n},Y_{n,ref}\right),Y_{n}w_{b}\left(K_{n},Y_{n,ref}\right) \end{array}\\ \rho (X^{T}w_{c}\left(X_{n},Y_{n,ref}\right),Y_{n}w_{d}\left(X_{n},Y_{n,ref}\right)\\ \rho (X^{T}w_{c}\left(X_{n},Y_{n,ref}\right),{\bar{X}}_{n}w_{c}\left(X_{n},Y_{n,ref}\right) \end{array}\right]$$

To select the candidate stimuli from the obtained features of ${\hat{r}}_{n}$ , we employed a clustering method. The process begins with selecting the optimal number of clusters by utilizing Gaussian Mixture Models (GMM) and evaluating them with the Davies-Bouldin index (Setiawan and Kurniawan, 2023). Features are clustered with GMM for various numbers of clusters h , and the Davies-Bouldin index is calculated for each h . The number of clusters that yield the lowest Davies-Bouldin index is selected as the optimal number. Once the optimal number of clusters is identified, k-means++ clustering (Arthur and Vassilvitskii, 2006) is applied to the features. Next, the average of the features (correlation coefficients) within each cluster is computed and the cluster with the highest value is selected. This cluster encompasses the candidate stimulus frequencies.

In the second stage, the correlation coefficients for each candidate stimulus (indexed by k ) are summed up resulting in a vector denoted by T including m candidate stimulus, as shown in Equation (10):


(10) $$T\left(k\right)=\overset{5}{\underset{s=1}{\Sigma }}{\hat{r}}_{k}\left(s\right),\;k=1,2,\ldots ,m$$

Subsequently, the Pearson’s correlation coefficients between the single-trial test signal $X\in {\mathbb{R}}^{N_{c}*N_{s}}$ and the averaged training trials for the $m^{th}$ visual stimulus, ${\bar{X}}_{m}\in {\mathbb{R}}^{N_{c}*N_{s}}$ , are computed as described in Equation (11):


(11) $$D\left(k\right)=\rho \left(X^{T}W_{t},{\bar{X}}_{k}^{T}W_{t}\right),\;k=1,2,\ldots ,m$$

where $W_{t}=\left[w_{t1},\;w_{t2},\ldots ,w_{tm}\right]$ is the concatenated TRCA spatial filters computed in the training step. Further improvement can be achieved by integrating spatial filters, as demonstrated in the study (Nakanishi et al., 2017). Then, the overall correlation coefficients for the $m^{th}$ candidate SSVEP stimulus are computed by summing the values of D and T which is defined as C .

Finally, the target frequency $f_{t}$ , representing the SSVEP stimulus with the highest correlation coefficient, is defined as the maximum element of the vector $C_{k}$ , as shown in Equation (12):


(12) $$f_{t}=\underset{k}{\mathrm{argmax}}C_{k},\;k=1,2,\ldots ,m$$

**Figure 1:**
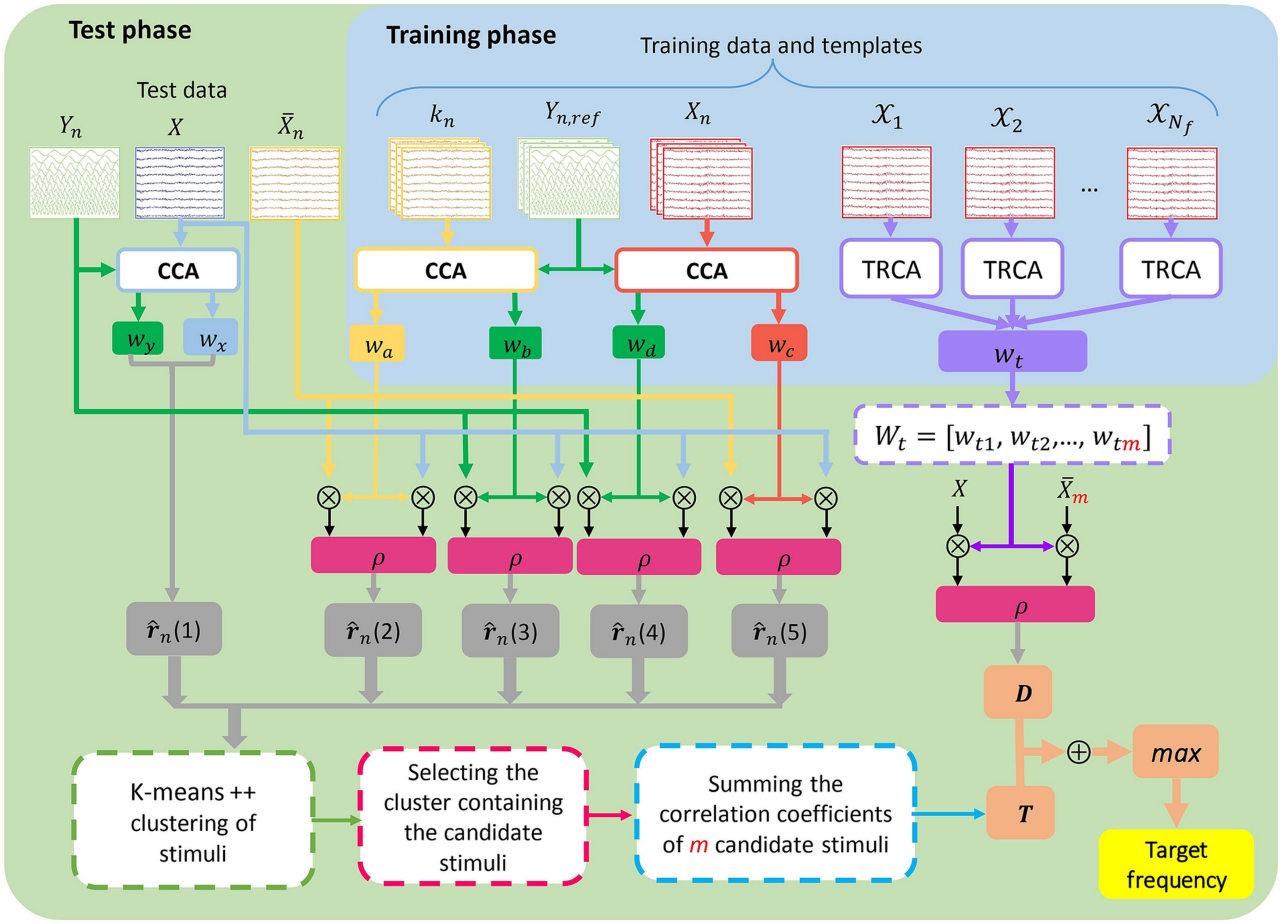
Block diagram of the proposed H-TRCCA method. In the training phase, CCA-based spatial filters (*w_a_*, *w_b_*, *w_c_*, *w_d_*) and a TRCA-based filter (*w_t_*) are extracted. In the test phase, canonical and Pearson correlations are computed and clustered using k-means to select candidate stimuli. The final target frequency is identified by summing the Pearson correlation coefficients (*D*) and the candidate correlation values (*T*), and selecting the stimulus with the highest overall correlation coefficient.

The flowchart shown in Figure 1 depicts the process of SSVEP target detection using the proposed H-TRCCA method.

### 2.5 Performance criteria

In evaluating SSVEP-based BCI systems, two crucial criteria are classification accuracy and ITR (Wolpaw et al., 1998). The performance of the H-TRCCA in frequency detection and its comparison with competitive techniques are evaluated using these criteria. Accuracy measures the system’s ability to make precise predictions, while ITR evaluates the efficiency of information transfer. The ITR is expressed by Equation (13):


(13) $$ITR\frac{\mathrm{bits}}{\min}=\left[\log_{2}N_{f}+P\log_{2}P+\left(1-P\right)\log_{2}\left(\frac{1-P}{N_{f}-1}\right)\right]\times \frac{60}{T_{w}}$$

where P represents the accuracy of target stimuli recognition, and $T_{w}$ is the required time during each epoch to perform the frequency recognition.

This study utilized MsetCCA-R (Wong et al., 2020b), SSCOR (Kumar and Reddy, 2019), TRCA (Nakanishi et al., 2017), and msTRCA (Wong et al., 2020a) as competing methods. In the case of msTRCA, the number of neighboring frequencies was set to two, whereas for MsetCCA-R and H-TRCCA, the $N_{h}$ parameter of the artificial sine-cosine signals was set to five. Additionally, all methods utilized five frequency bands. A comparative assessment was undertaken to analyze the efficacy of these techniques in classifying stimulus frequencies for each dataset. The recognition performance of the algorithms was evaluated by calculating accuracy and ITRs using a leave-one-block-out cross-validation approach. For Dataset I, five blocks of SSVEP signals were used for training, with one block reserved for testing, and this process was repeated six times. Similarly, for Dataset II, three blocks were used for training and one block for testing, and this was repeated four times. Specifically, for a dataset with B blocks of EEG signals, ( B -1) blocks were selected for training, while the remaining block was used for testing, and this was repeated B times.

### 2.6 Experimental setup

The experimental results and statistical analyses were obtained using Matlab 2020b (The MathWorks, Inc., Natick, MA, USA) and SPSS Statistics 27 (IBM, Armonk, NY, USA) on an ASUS PC with a 12th generation Intel(R) Core (TM) i5-1250H @ 2.50 GHz processor, 16 GB of RAM, and a 64-bit Windows 10 OS.

## 3 Results

### 3.1 Target detection performance

**Figure 2:**
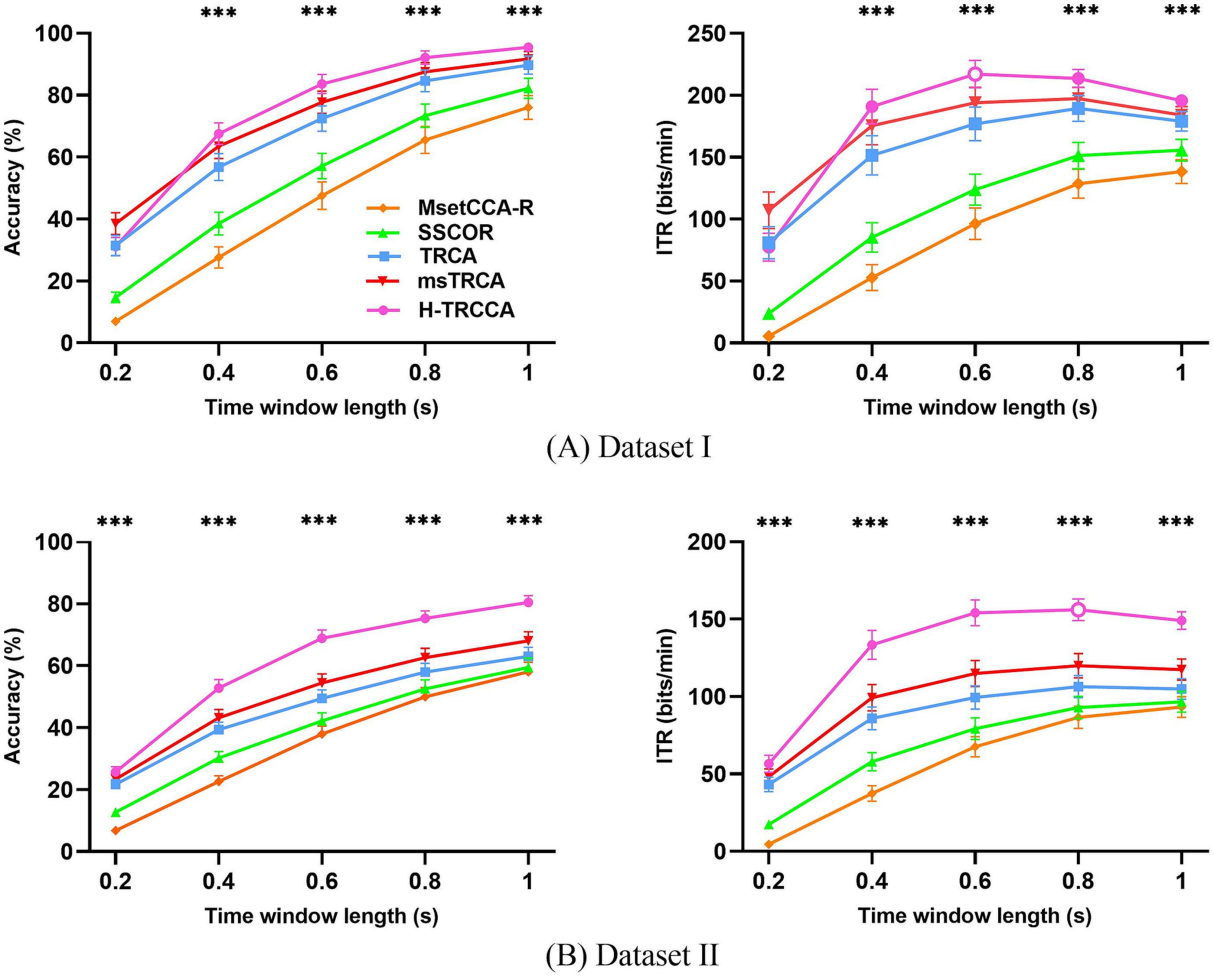
Comparing the average accuracy and ITR achieved across all subjects using different methods for **(A)** Dataset I (five training trials) and **(B)** Dataset II (three training trials), with varying data lengths. The error bars denote the standard error. The asterisks in the Figure indicate statistical significance, as determined by one-way repeated-measures ANOVA. The symbol *** denotes the significance levels (*p* < 0.001), indicating significant differences between the five algorithms. O indicates the highest ITR value.

Figure 2 provides a comprehensive comparison of accuracy and ITR for different data lengths ( $T_{w}$ ) corresponding to various target detection techniques on two datasets. The $T_{w}$ ranges from 0.2 s to 1 s with an interval of 0.2 s. In Figure 2A, the upper row illustrates the accuracy and ITR for Dataset I, while Figure 2B‘s lower row presents the accuracy and ITR results for Dataset II. A one-way repeated-measures ANOVA was employed to evaluate the comparability of performance across the different techniques for both datasets. In Dataset I, H-TRCCA exhibited a notable improvement of 36.04% over MsetCCA-R, 26.50% over SSCOR, 11.16% over TRCA, and 5.92% over msTRCA at $T_{w}=$ 0.6 s. Similarly, in Dataset II, H-TRCCA demonstrated enhancements of 29.06% over MsetCCA-R, 24.80% over SSCOR, 17.49% over TRCA, and 12.53% over msTRCA at $T_{w}=$ 0.6 s. Significant differences were observed between H-TRCCA and the other four methods in Dataset I for all $T_{w}$ values except 0.2 s and in Dataset II for all different $T_{w}$ values (*p* < 0.001). Regarding the ITR results, optimal ITR values were mostly achieved with medium data lengths for both datasets. In Dataset I, the highest ITR values were 138.42 bits/min ( $T_{w}=$ 1 s) for MsetCCA-R, 155.80 bits/min ( $T_{w}=$ 1 s) for SSCOR, 189.50 bits/min ( $T_{w}=$ 0.8 s) for TRCA, 197.39 bits/min ( $T_{w}=$ 0.8 s) for msTRCA, and 217.41 bits/min ( $T_{w}=$ 0.6 s) for H-TRCCA. In Dataset II, the highest ITR values were 93.32 bits/min ( $T_{w}=$ 1 s) for MsetCCA-R, 96.64 bits/min ( $T_{w}=$ 1 s) for SSCOR, 106.47 bits/min ( $T_{w}=$ 0.8 s) for TRCA, 119.84 bits/min ( $T_{w}=$ 0.8 s) for msTRCA, and 156.34 bits/min ( $T_{w}=$ 0.8 s) for H-TRCCA. A significant difference was observed between the H-TRCCA algorithm and the other four algorithms in Dataset I for all $T_{w}$ values except 0.2 s and in Dataset II for all different $T_{w}$ values (*p* < 0.001).

A one-way repeated-measures ANOVA was performed to evaluate the effects of different target detection methods on accuracy and ITR across $T_{w}$ for Dataset I and Dataset II. The results indicate that the choice of target detection method significantly affects both accuracy and ITR, with variations observed depending on the $T_{w}$ and dataset. In general, higher accuracy was associated with longer time windows, while optimal ITR values were found at medium time windows ( $T_{w}=$ 0.6–0.8 s). Detailed statistical comparisons are presented in Table 1.

**Table 1:** Comparison of the statistical results of the one-way repeated-measures ANOVA for accuracy across different time windows in Dataset I and Dataset II.

Evaluation metrics	Dataset	Time Window (s)	F (df1, df2)	*p*-value	Partial $\eta^{2}$
Accuracy	Dataset I	0.4	*F* (2.04, 69.63) = 199.08	<0.001	0.85
		0.6	*F* (1.88, 64.07) = 124.92	<0.001	0.78
		0.8	*F* (1.91, 65.21) = 59.70	<0.001	0.63
		1	*F* (2.08, 70.89) = 32.31	<0.001	0.48
	Dataset II	0.4	*F* (1.78, 123.39) = 281.05	<0.001	0.80
		0.6	*F* (2.33, 161.24) = 301.23	<0.001	0.81
		0.8	*F* (2.21, 153.05) = 224.56	<0.001	0.76
		1	*F* (1.79, 123.66) = 177.71	<0.001	0.72
ITR	Dataset I	0.4	*F* (1.71, 58.35) = 157.55	<0.001	0.82
		0.6	*F* (2.01, 68.44) = 153.70	<0.001	0.81
		0.8	*F* (2.06, 70.15) = 82.28	<0.001	0.70
		1	*F* (2.20, 74.88) = 45.10	<0.001	0.57
	Dataset II	0.4	*F* (1.49, 133.33) = 191.48	<0.001	0.73
		0.6	*F* (1.92, 132.64) = 255.95	<0.001	0.78
		0.8	*F* (2.17, 150.20) = 236.44	<0.001	0.77
		1	*F* (1.74, 120.04) = 196	<0.001	0.74

The table presents the F-statistic (F), degrees of freedom (df), *p*-values, and partial eta squared (η^2), indicating the effect of different target detection methods on accuracy.

**Figure 3:**
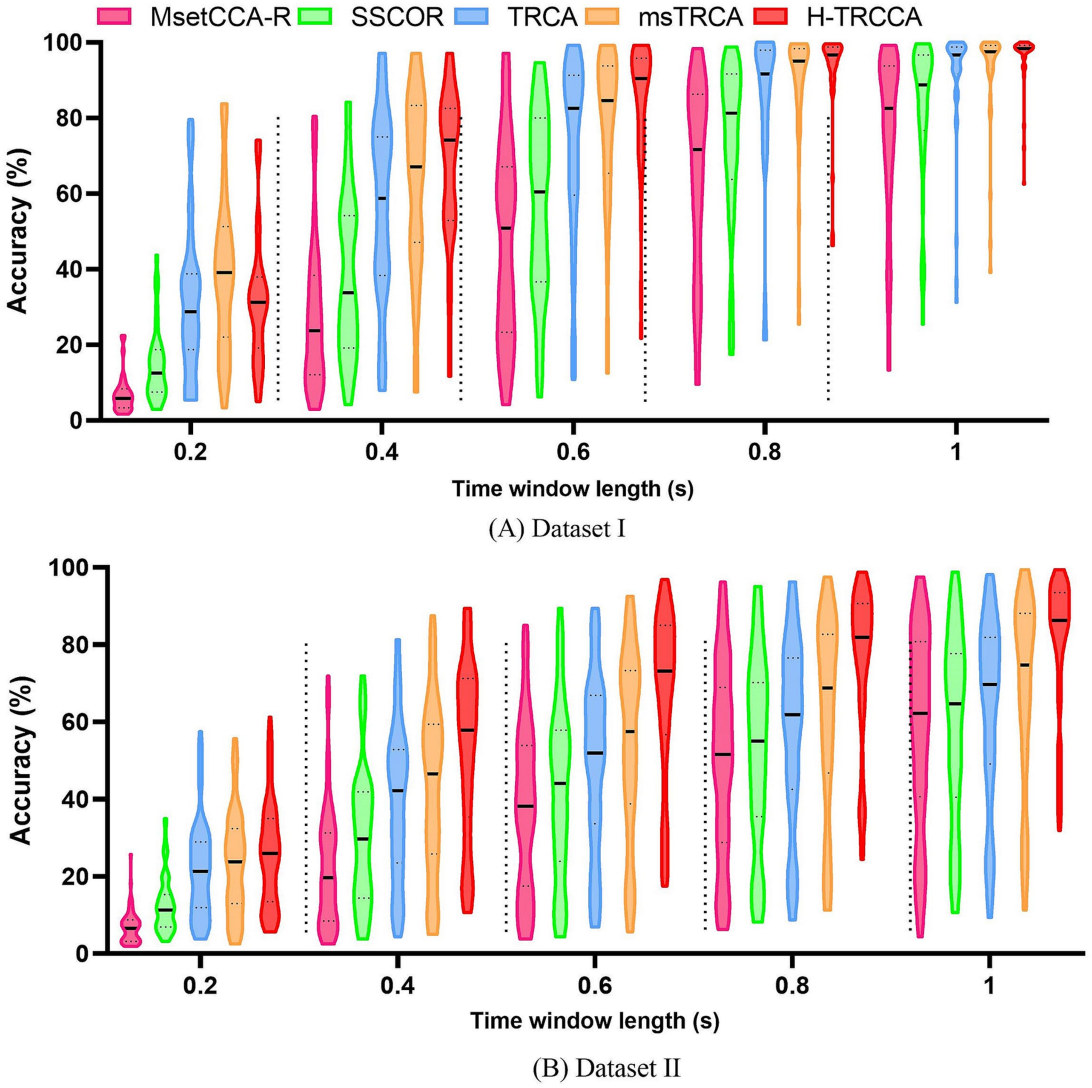
Violin plots showing the accuracy distributions achieved by the five algorithms for varying data lengths on two datasets: **(A)** Dataset I and **(B)** Dataset II. Each violin plot features a thick black line indicating the median, with two additional black lines on either side representing the interquartile ranges (25 and 75% percentiles).

Figure 3 showcases violin plots that illustrate the probability density of accuracy for five algorithms applied to both datasets. These plots provide a visually intuitive representation of the distribution of quantitative data. Analysis of the plots reveals that the violin plots for H-TRCCA (shown in red) generally exhibit higher median values and more concentrated distributions in both datasets. This suggests that H-TRCCA consistently achieves superior and stable classification performance across different subjects, outperforming the competing algorithms. Figure 3B shows a more dispersed distribution for all methods than Figure 3A due to the larger number of subjects in Dataset II.

### 3.2 The effect of the number of training trials on detection performance

**Figure 4:**
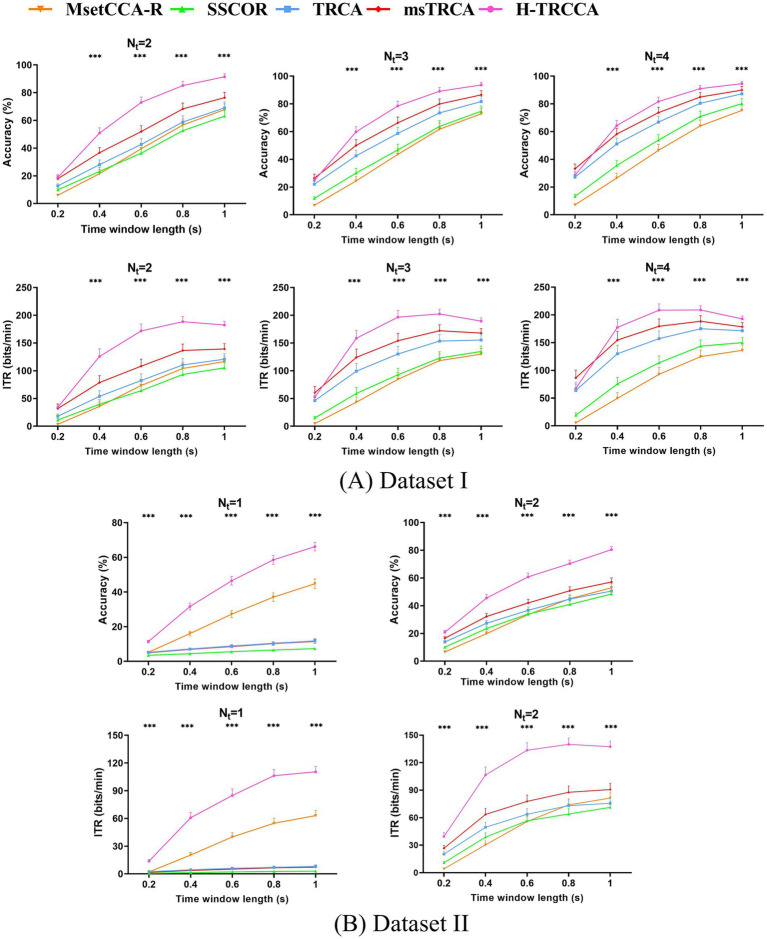
Comparison of the average accuracy and ITR results for different numbers of training trials and data lengths: **(A)** Dataset I with 2 to 4 training trials, and **(B)** Dataset II with 1 to 2 training trials. Statistical significance was determined using a one-way ANOVA, with *** indicating *p* < 0.001.

Given the limitations of dataset collection in BCI applications, it is crucial to evaluate performance using a small number of $N_{t}$ instances when there is insufficient data available (Lan et al., 2023; Du et al., 2024). The primary objective of H-TRCCA is to accurately classify SSVEP frequencies with minimal individual training data. The frequency recognition performance in the H-TRCCA is influenced by both the construction of template signals using individual training data and the number of $N_{t}$ . Figure 4 compares the accuracy and ITR of state-of-the-art methods with H-TRCCA across different numbers of $N_{t}$ and $T_{w}$ in Dataset I and Dataset II. Based on Figure 4, the H-TRCCA demonstrates superior classification performance compared to other methods across various data lengths and numbers of training data. This superiority is especially evident when there is limited calibration data available. (i.e., only one or two trials). Moreover, Figure 4 demonstrates that increasing the $N_{t}$ results in increased classification accuracy and ITR. Furthermore, as $N_{t}$ decreases for both Dataset I and Dataset II, the difference in accuracy and ITR between H-TRCCA and competitive algorithms widens for all data lengths. To further investigate these effects, a two-way repeated measures ANOVA (method × block) confirmed a significant main effect of blocks. For the Dataset I, the analysis yielded *F* (1.18, 40.34) = 289.11, *p* < 0.001, with Greenhouse–Geisser correction applied. Similarly, a significant effect was observed for Dataset II, *F*(1.07, 74.23) = 331.55, *p* < 0.001, also adjusted using the Greenhouse–Geisser correction. Additionally, the analysis revealed a significant interaction effect between the methods and blocks for Dataset I, *F* (3.45, 117.50) = 46.45, *p* < 0.001, and for Dataset II, *F* (1.77, 122.18) = 115.47, *p* < 0.001, both adjusted using the Greenhouse–Geisser correction.

Tables 2, 3 present the numerical classification accuracy and ITR of five methods across different $N_{t}$ values, while keeping $T_{w}$ fixed at 0.6 s. Additionally, the tables include the results of a one-way repeated-measures ANOVA analysis for these methods. The training block range for Dataset I was from 2 to 5, and for Dataset II, it was from 1 to 3. The one-way repeated-measures ANOVA revealed a statistically significant difference (*p* < 0.001) among the compared algorithms for all training block numbers in both datasets. The analysis highlights the effectiveness of the H-TRCCA method, particularly with limited training trials. The results indicate that H-TRCCA achieves accuracies comparable to msTRCA with significantly fewer training trials. Specifically, for Dataset I with $N_{t}$ ≥ 3 and Dataset II with $N_{t}$ ≥ 2, H-TRCCA performs comparably to msTRCA at $N_{t}=$ 5, demonstrating its efficiency with less training data.

**Table 2:** Comparison of accuracy and ITR between five algorithms with different numbers of training blocks in Dataset I, using a data length of 0.6 s.

Methods	Accuracy and ITR with different numbers of training blocks							
	Accuracy (%)				ITR (bits/min)			
	2	3	4	5	2	3	4	5
MsetCCA-R	39.54	43.55	46.66	47.57	73.25	84.62	92.81	96.43
SSCOR	36.34	46.78	53.85	57.11	64.01	92.67	113.59	123.83
TRCA	42.61	58.69	66.80	72.45	82.40	130.18	157.09	177.07
msTRCA	51.96	66.29	73.64	77.69	108.17	154.08	179.58	194.07
H-TRCCA	**73**	**78.5**	**81.64**	**83.61**	**171.73**	**196.54**	**208.52**	**217.41**
*p*-value	<0.001	<0.001	<0.001	<0.001	<0.001	<0.001	<0.001	<0.001

The best values for each of the methods are highlighted in bold.

**Table 3:** Comparison of accuracy and ITR between five algorithms with different numbers of training blocks in Dataset II, using a data length of 0.6 s.

Methods	Accuracy and ITR with different numbers of training blocks					
	Accuracy (%)			ITR (bits/min)		
	1	2	3	1	2	3
MsetCCA-R	27.22	33.57	37.92	39.97	55.79	67.62
SSCOR	5.56	34	42.18	2.07	56.61	79.26
TRCA	8.86	36.64	49.47	6.03	63.70	99.47
msTRCA	8.67	42	54.45	5.47	77.80	114.89
H-TRCCA	**46.25**	**60.70**	**66.98**	**89.05**	**132.92**	**154.72**
*p*-value	<0.001	<0.001	<0.001	<0.001	<0.001	<0.001

The best values for each of the methods are highlighted in bold.

**Figure 5:**
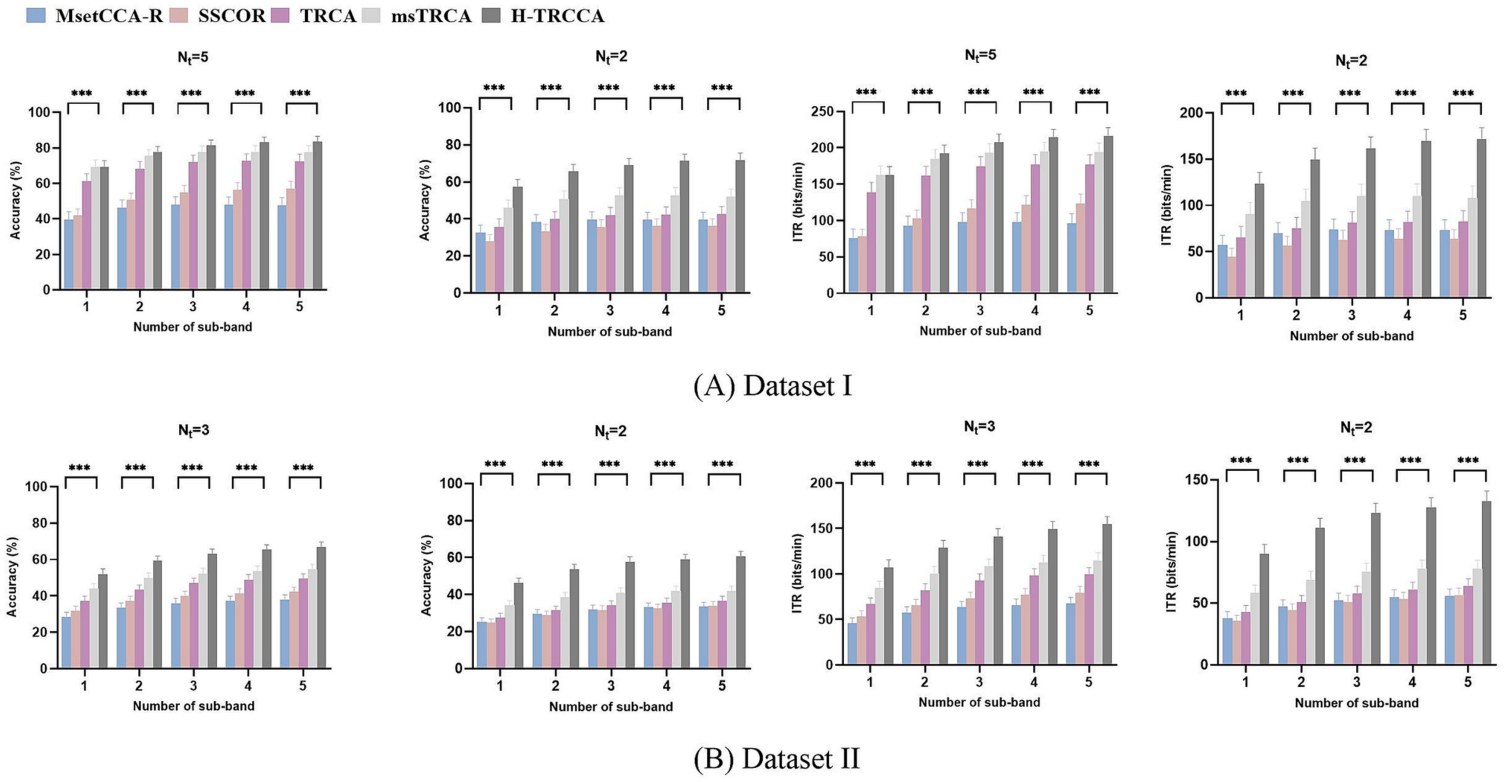
Bar charts depicting the accuracy and ITR for all algorithms across different numbers of sub-bands. The asterisks on the chart signify significant differences among the five techniques, as specified by one-way repeated-measures ANOVA (^***^p < 0.001). The labels **(A)** and **(B)** denote the results for Dataset I and Dataset II, respectively.

In this study, we examined the influence of the number of sub-bands on the performance of the H-TRCCA and the compared algorithms. Figure 5 demonstrates the algorithm’s performance across different numbers of sub-bands (ranging from 1 to 5) for both Dataset I and Dataset II, along with two different $N_{t}$ values with $T_{w}$ set at 0.6 s. The H-TRCCA consistently demonstrated superior accuracy and ITRs across the various sub-bands. To further assess and compare these techniques, a one-way repeated-measures ANOVA was conducted. Comparing cases with sufficient training trials ( $N_{t}=$ 5 for Dataset I and $N_{t}=$ 3 for Dataset II) and cases with insufficient calibration data ( $N_{t}=$ 2) for both datasets showed a significant disparity in accuracy and ITR between the H-TRCCA approach and the other four approaches, as indicated by the results of the statistical analysis.

### 3.3 Effect of the number of EEG channels and training blocks on detection performance

**Figure 6:**
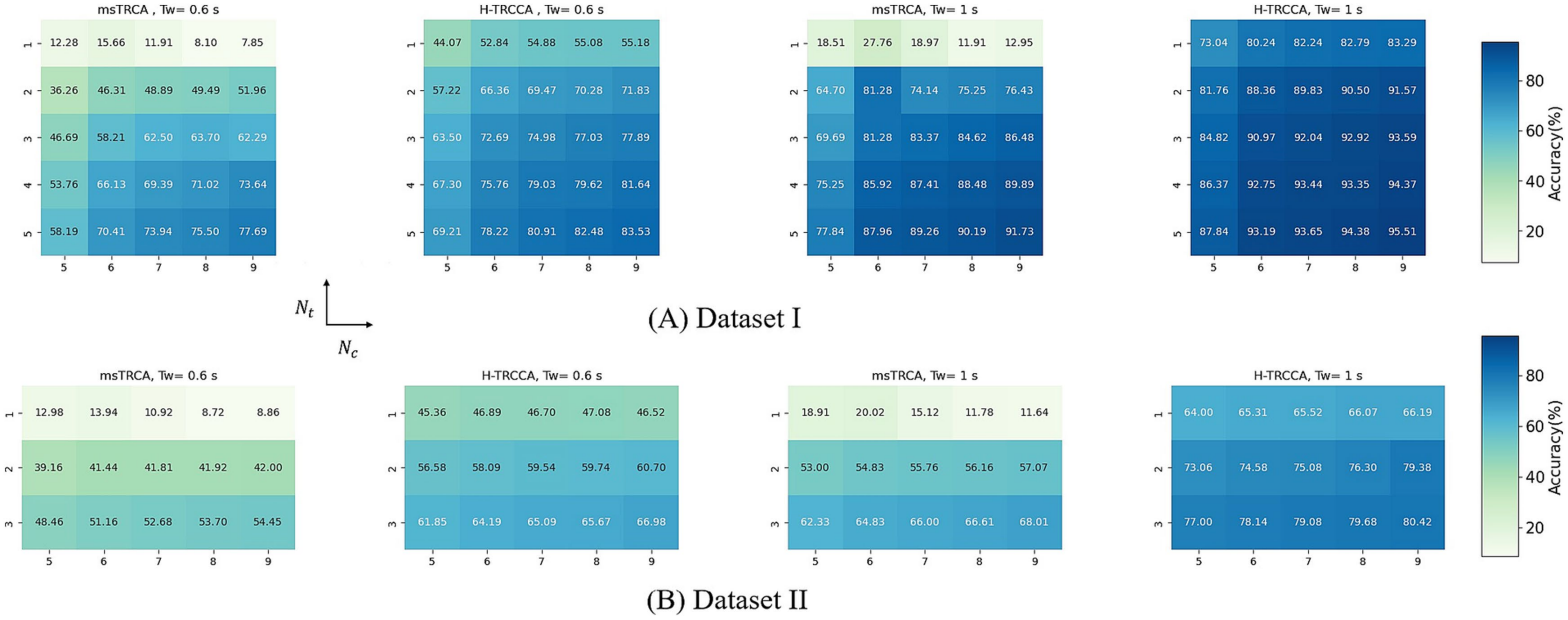
Comparison of frequency recognition accuracy between H-TRCCA and msTRCA using different numbers of training trials (i.e., *N_t_*) and numbers of EEG channels (i.e., *N_c_*) for **(A)** Datasets I and **(B)** Datasets II.

The number of channels can have a significant impact on determining the accuracy. We investigated the impact of channel count (ranging from 5 to 9) on the performance of algorithms (H-TRCCA and msTRCA) across different numbers of training trials in Dataset I and Dataset II. Regardless of the $T_{w}$ , the H-TRCCA consistently outperforms the msTRCA method in terms of different numbers of $N_{t}$ and EEG channels, in both datasets. As depicted in Figure 6, under two trial training conditions, H-TRCCA achieved an accuracy of 20.93 to 15.41% higher than msTRCA in Dataset I. The highest average accuracies with H-TRCCA, at data lengths of 0.6 s and 1 s, reached 71.83 and 91.57%, respectively. In Dataset II, H-TRCCA outperformed msTRCA by 17.42 to 22.31%, with the highest accuracies at data lengths of 0.6 s and 1 s reaching 57.07 and 79.38%, respectively. These findings demonstrate H-TRCCA’s superior performance with significantly less training data compared to the msTRCA. These results demonstrate that H-TRCCA consistently achieves higher accuracy than msTRCA across all cases. The accuracy difference is more significant at lower values of $N_{t}$ and $N_{c}$ , decreasing as these values increase, yet H-TRCCA maintains better performance throughout.

**Figure 7:**
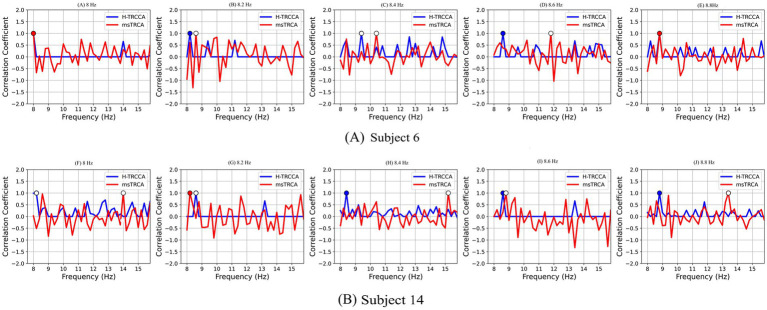
Correlation coefficients were obtained using H-TRCCA and msTRCA for five test trials (data length: 0.6 s) from two randomly selected subjects: **(A)** Subject 6 and **(B)** Subject 14. The recognition results are shown as blue and red circles for H-TRCCA and msTRCA, respectively. Hollow circles indicate incorrect detections, while solid ones indicate accurate detections.

Figure 7 illustrates the normalized correlation coefficients of forty stimuli obtained by H-TRCCA and msTRCA algorithms, using parameters $N_{t}=$ 2, $T_{w}=$ 0.6 s, and $N_{c}=$ 9 for five test trials corresponding to frequencies of 8, 8.2, 8.4, 8.6, and 8.8 Hz. Data were acquired from two randomly selected subjects (Subject 6 and Subject 14) from Dataset I. Insufficient training trials weaken the spatial filters, reducing their effectiveness and causing high peaks at non-target frequencies. Consequently, this decreases the accuracy of target frequency identification, as the algorithms rely on the highest correlation coefficient. As demonstrated in Figure 7, H-TRCCA exhibits reduced fluctuation in feature values at non-target frequencies compared to msTRCA. This reduced fluctuation can be attributed to the clustering algorithm used in the test phase, which effectively suppresses frequencies that are not candidates for target detection. The effectiveness of H-TRCCA is further illustrated in Figure 7 (Graphs B, D, H, I, and J), where the H-TRCCA method consistently outperforms in detecting the target frequency by minimizing non-target peaks at 8.2, 8.4, 8.6, and 8.8 Hz, respectively. Overall, H-TRCCA develops more effective spatial filters than msTRCA with a limited number of training trials, thereby minimizing interference from non-target frequencies.

### 3.4 Ensemble version of the methods

To comprehensively assess the efficacy of the proposed method, here, we implemented ensemble versions for the four algorithms. The aim of the ensemble spatial filter, as proposed by Nakanishi et al. (2017), is to integrate spatial filters from all stimulation targets to improve frequency recognition. This approach assumes that filters in identical frequency bands across various targets share similarities, and combining them can enhance spatial filtering efficacy. To create an ensemble spatial filter, these filters $w_{1},\;w_{2},\ldots ,w_{N_{f}}$ are concatenated, resulting in $W=\left[w_{1},\;w_{2},\ldots ,w_{N_{f}}\right]$ . Consequently, the calculation of the correlation coefficient for the $n^{th}$ target frequency is modified by Equation (14):


(14) $$S_{n}=\rho \left(X^{T}W,{\bar{X}}_{n}^{T}W\right),\;n=1,2,\ldots ,N_{f}$$

**Figure 8:**
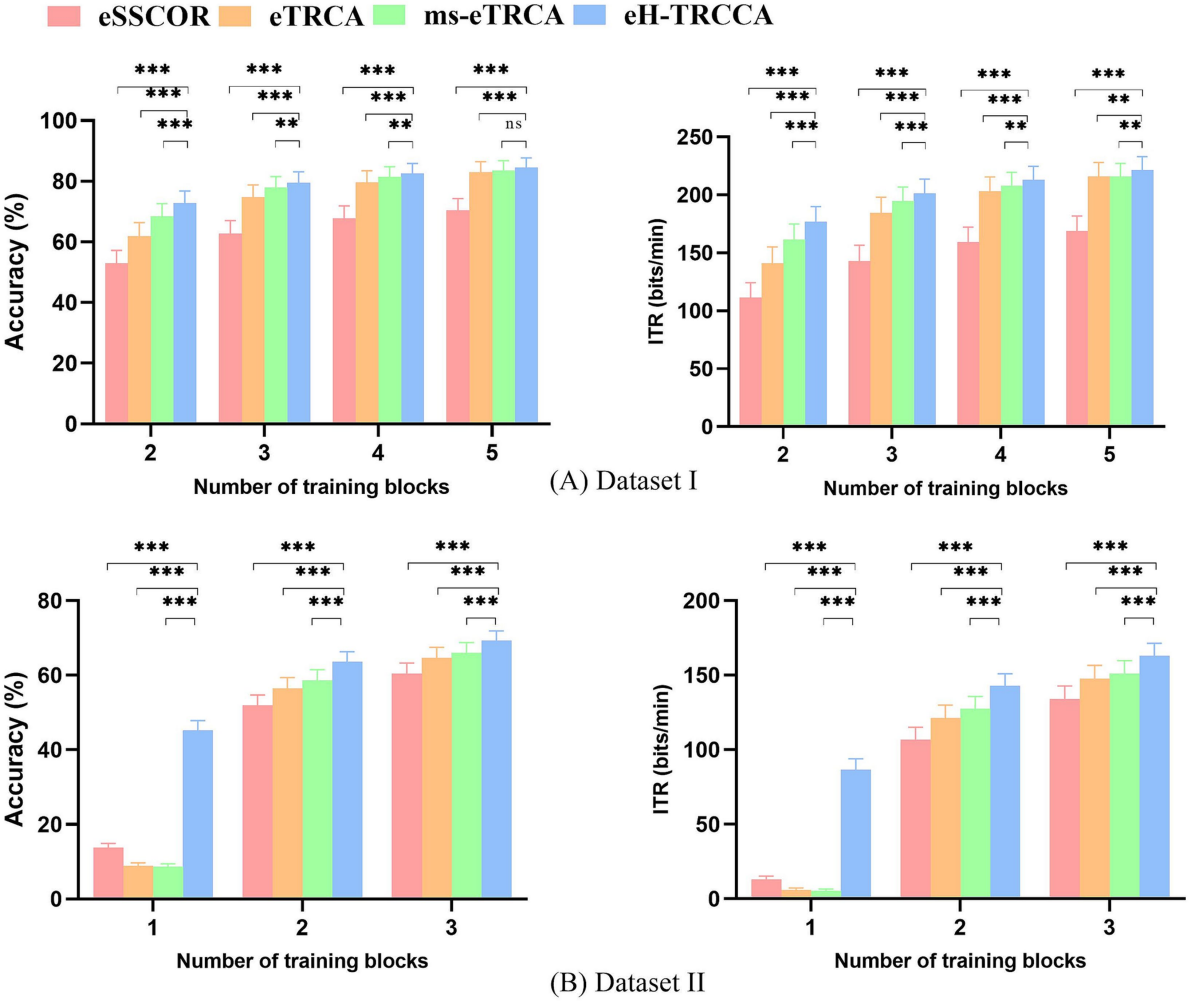
Comparison of average recognition accuracy and ITR results between ensemble versions of algorithms for different numbers of training trials in **(A)** Dataset I and **(B)** Dataset II. The asterisks in the subfigures indicate significant differences between the two methods, determined by paired t-tests (^*^p < 0.05, ^**^p < 0.01, ^***^p < 0.001).

In Figure 8, a comparison of accuracy and ITR for ensemble-based methods across different numbers of training trials and data length 0.6 s on both Dataset I and Dataset II is presented. The upper row of Figure 8A shows the accuracy and ITR for Dataset I, while the lower row of Figure 8B presents the accuracy and ITR results for Dataset II. In most conditions, the ensemble version of the proposed approach (eH-TRCCA) demonstrated superior performance compared to the ensemble versions of competing methods, with higher accuracy. For example, in Dataset I with $N_{t}=$ 2, eH-TRCCA outperformed ms-eTRCA by 4.25%, eTRCA by 10.88%, and eSSCOR by 19.86%. Similarly, in Dataset II, eH-TRCCA achieved accuracy improvements of 5, 9.20, and 11.79% over ms-eTRCA, eTRCA, and eSSCOR, respectively. Furthermore, eH-TRCCA had an average ITR of 176.73 bits/min, whereas ms-eTRCA achieved 161.55 bits/min, eTRCA achieved 141.09 bits/min, and eSSCOR achieved 111.50 bits/min for $N_{t}=$ 2 on average in Dataset I. In Dataset II, for $N_{t}=$ 2, eH-TRCCA reached the highest ITR at 142.72 bits/min, while ms-eTRCA, eTRCA, and eSSCOR attained maximum ITRs of 127.33 bits/min, 121.33 bits/min, and 106.93 bits/min, respectively. Paired t-tests show that eH-TRCCA’s accuracy and ITR are significantly better than those of other ensemble methods in most cases, as indicated in Figures 8A,B.

## 4 Discussion

In practical SSVEP-based BCI systems, the need for extensive training trials becomes more evident as the number of SSVEP targets increases, often requiring more than forty stimuli (Sun et al., 2021). Insufficient training data can significantly reduce SSVEP frequency recognition performance (Wong et al., 2020b). Consequently, the time-consuming and fatiguing nature of the training process for the BCI user may hinder the collection of a substantial number of training trials (Zerafa et al., 2018). Hence, subject-specific training methods, which necessitate training with a large number of individualized data to enhance SSVEP frequency detection, do not seem to constitute an effective solution. However, to meet the practical needs of applications, there remains a need to improve the accuracy of frequency detection performance by reducing training epochs. Most subject-specific training techniques in SSVEP-based BCI domains have developed spatial filters considering the correlation between the training data and the artificial signal (Yuan et al., 2022) or the individual template (Wei et al., 2020), as well as the relationship across training trials (Nakanishi et al., 2017; Kumar and Reddy, 2019). This study introduced a novel approach called H-TRCCA, which combines the strengths of both CCA and TRCA. CCA assesses the fundamental and harmonic oscillations of SSVEPs by measuring the correlation between EEG data and reference signals, while TRCA enhances the reproducibility of task-related components across trials by maximizing covariance. Both the oscillatory features of SSVEPs and task-related components are taken into account for accurate SSVEP detection (Lan et al., 2023). Methodologically, H-TRCCA presents several notable differences compared to state-of-the-art techniques. Firstly, subject-specific training methods like msTRCA, TRCA, and SSCOR often perform poorly when training data is insufficient. This issue arises because the reliability of the covariance matrix decreases, leading to less effective spatial filters (Sun et al., 2021). One contributing factor to this issue is the absence of artificial reference signals. To address this challenge and improve frequency detection accuracy with limited training data, the H-TRCCA method utilizes training data, individual templates, and reference signals. This method enhances frequency recognition by computing five correlation coefficients from various filter-template combinations and integrating them intelligently for better detection accuracy. As shown in Figure 7, H-TRCCA creates more effective spatial filters compared to msTRCA methods when calibration data is insufficient. Furthermore, recent studies have highlighted that classification features based on a single correlation coefficient exhibit poorer performance compared to an ensemble of multiple correlation coefficients (Nakanishi et al., 2017; Kumar and Reddy, 2019). To improve performance, H-TRCCA takes a different approach compared to the other four algorithms. Instead of utilizing individual spatial filters for each stimulus, H-TRCCA concatenates the spatial filters of candidate stimuli, thereby creating a unified spatial filter. Additionally, employing spatial filters on selected candidate stimuli reduces fluctuations and effectively suppresses non-target frequencies. Therefore, utilizing features selected through the k-means++ algorithm can significantly enhance SSVEP detection performance, as illustrated in Figure 7. Lastly, the H-TRCCA aims to construct a robust and accurate classifier by combining the correlation coefficients obtained from CCA-based spatial filters with those obtained from TRCA-based integrated candidate stimulus spatial filters for each candidate stimulus. Compared to the other methods examined in this study, H-TRCCA shows greater potential in addressing the issue of insufficient training data.

The effectiveness of H-TRCCA has been validated across diverse conditions using two publicly available datasets. A comparison of results from both datasets consistently shows that H-TRCCA outperforms state-of-the-art approaches. This superiority was consistently observed across different numbers of training blocks and sub-bands, as well as varying numbers of channels, which are important parameters in target recognition. These results are depicted in Figures 2, 4–6. Specifically, Figure 2A demonstrates the superior performance of the H-TRCCA over msTRCA and TRCA on Dataset I, specifically for data lengths of 0.6 s. The accuracy achieved by the H-TRCCA is 7.19% higher than that of msTRCA and an impressive 19.05% higher than that of TRCA. Based on previous studies, msTRCA has been shown to significantly outperform TRCA in handling insufficient calibration data (Wong et al., 2020a; Sun et al., 2021). Additionally, msTRCA demonstrates substantial improvements over TRCA in various benchmark datasets (Sun et al., 2021). The findings of this study suggest that H-TRCCA outperforms both TRCA and msTRCA in frequency detection, particularly when there is inadequate training data. As illustrated in Figure 4A, H-TRCCA achieves an accuracy of 77.89% with only two training trials, whereas msTRCA and TRCA require five training trials to reach accuracies of 77.69 and 72.45% respectively, on Dataset I. This demonstrates the superior performance of H-TRCCA in scenarios with limited training data allowing for a significant reduction in calibration time while maintaining performance. This study also explored the impact of the number of channels on different numbers of training data. The results shown in Figure 6 show that H-TRCCA has superior performance in frequency detection compared to the msTRCA, especially when the number of channels is small and a limited amount of training data is available. Reducing the number of channels in a real-world BCI system lowers implementation costs, enhances user experience, and improves overall comfort. To further investigate, we conducted a comparison between the ensemble version of H-TRCCA and the ensemble versions of state-of-the-art approaches. The results demonstrate that the ensemble H-TRCCA consistently outperforms the ensemble versions of other methods, highlighting its robustness and effectiveness. Moreover, as the number of training trials decreases, the performance difference between eH-TRCCA and the other four methods increases. Notably, the proposed method demonstrated a significantly greater improvement in practical SSVEP-based BCI systems (e.g., Dataset II) compared to state-of-the-art methods under controlled laboratory conditions (represented by Dataset I). This suggests that the proposed method may perform more effectively in real-world scenarios where noise and variabilities are present.

In EEG-based BCIs, computational efficiency is critical for real-time applications. In this study, we compared the computational times of H-TRCCA and TRCA methods. During the spatial filter training phase, H-TRCCA took 0.6287 s for Dataset I and 0.4724 s for Dataset II, while TRCA took 0.5240 s and 0.3754 s, respectively. After the training phase, the average time per window for target recognition was 0.0846 s for Dataset I and 0.0886 s for Dataset II with H-TRCCA, compared to 0.0247 s for Dataset I and 0.0239 s for Dataset II with TRCA. Although the proposed method required slightly more time for testing compared to TRCA, the difference was only a few milliseconds, which does not affect the computational speed of target detection. Therefore, both methods can be applied to online experiments.

While the H-TRCCA method has shown promising performance in detecting SSVEP-based BCI compared to other approaches in the field, there is still potential for further improvement. One limitation of H-TRCCA is its focus on a fixed data length, which may not be optimal in all scenarios. To overcome this limitation, future work can explore a dynamic window approach, allowing for dynamic adjustment of the data length collected for each trial. Additionally, in the proposed method, instead of using standard TRCA, advanced methods like msTRCA (Wong et al., 2020a), TRCA-R (Wong et al., 2020b), and scTRCA (Sun et al., 2021) can be employed. Combining these methods may offer improved performance compared to H-TRCCA, especially when there is insufficient calibration data.

## 5 Conclusion

In this study, we introduced the Hybrid task-related component and canonical correlation analysis (H-TRCCA), a novel method designed to enhance the performance of SSVEP-based BCIs, especially when training data is limited. H-TRCCA effectively combines the strengths of CCA and TRCA to create spatial filters that improve frequency detection accuracy and reduce the need for extensive training trials. The results show that H-TRCCA consistently surpasses state-of-the-art methods, including MsetCCA-R, SSCOR, TRCA, and msTRCA, even with a limited number of trials. The method’s effectiveness is supported by comprehensive evaluations using two public datasets: Dataset I (benchmark dataset) and Dataset II (BETA dataset). The results highlight H-TRCCA’s capability to achieve high classification performance despite constrained training data.

## Data Availability

The original contributions presented in the study are included in the article/supplementary material, further inquiries can be directed to the corresponding author.
